# “I miss seeing the kids!”: Australian teachers’ changing roles, preferences, and positive and negative experiences of remote teaching during the COVID-19 pandemic

**DOI:** 10.1007/s13384-022-00565-w

**Published:** 2022-08-30

**Authors:** Penny Van Bergen, Emily Daniel

**Affiliations:** 1 Macquarie School of Education, Macquarie University, Sydney, Australia; 2 School of Education, University of Wollongong, Wollongong, Australia; 3 School of Psychological Science, Macquarie University, Sydney, Australia

**Keywords:** Remote teaching, Online learning, Technology, COVID-19, Student–teacher relationships, Wellbeing

## Abstract

The COVID-19 pandemic has caused significant upheaval in schools in Australia and internationally. The aim of this study was to map Australian teachers’ positive and negative experiences during remote and online learning. Our study took place during the first COVID-19 wave, in the early stages of lockdown. Using an online instrument, we asked 210 primary and secondary teachers about changes in their teaching roles due to COVID-19. Responses were coded for positive and negative themes using inductive thematic analysis. The majority of teachers reported negative themes (88.6%), while half also reported positive themes (44.8%). Participants reported missing their students and struggling with excessive workload demands. They also experienced difficulties tracking student progress and felt worried for student wellbeing. Interestingly, concerns about technology were less common. Indeed, 19.1% enjoyed learning new online skills and integrating IT in new ways. Implications for student–teacher relationships, mental health, and future teaching are discussed.

COVID-19 has caused significant disruption to education internationally. For school teachers in Australia, a two-month lockdown during the first COVID-19 wave in March 2020 was followed by targeted state or city lockdowns during two subsequent infection waves and multiple smaller outbreaks (Storen & Corrigan, 2020; UNESCO, 2021). Across each lockdown, families were advised to keep children at home if they were able (Storen & Corrigan, 2020) while primary and secondary schools were asked to provide online and distance learning (Morrison, 2020). The rapid shift to remote and online teaching in Australia was echoed internationally, with unprecedented school closures in 191 countries affecting more than 90% of children worldwide (UNESCO, 2021).

Over the past two years a valuable corpus of educational research has emerged about the impacts of remote and online teaching on teachers and students. There is international evidence, for example, that teachers have experienced high levels of stress and exhaustion (e.g. Fray et al., 2022; MacIntyre et al. 2020; Silva et al., 2021). When viewed through the lens of the Job Demands-Resources model, these findings suggest that the specific “job demands” of teaching during COVID-19 often exceeded the structural and personal resources that teachers had available to manage those demands (Demerouti et al., 2001; Llorens et al., 2006).

While personal skills and qualities such as positive reframing techniques (MacIntyre et al. 2020) and emotional self-care (Sokal et al., 2020) appear to support the maintenance of positive job roles, these qualities may not fully mitigate the demands faced by teachers during this period of upheaval. Remote learning demands a different skillset to face-to-face teaching, including different pedagogies and greater use of technologies (Sokal et al., 2020), and self-efficacy for teaching (Haverback 2020) and for instructing remotely (Košir et al. 2020) are also valuable. In Slovenia, teachers who had lower self-efficacy for remote instruction during the first COVID-19 wave also had higher stress (Košir et al. 2020). Our own research shows that Australian teachers with lower instructional self-efficacy during lockdown and remote teaching had higher emotional exhaustion (blinded), while equivalent research with Canadian teachers during lockdown and remote teaching found that issues with technology, work-life balance, and under-resourcing all correlated with exhaustion (Sokal et al., 2020). Finally, structural resources available within the role are likely to be important to teachers’ perceptions of their roles during the move to remote learning. In a recent qualitative study, for example, Woltran et al. (2021) asked primary school teachers in Austria about the key professional challenges they faced in remote teaching. Alongside workload concerns, a lack of technical equipment and digital skills were identified.

There is also evidence, although mixed, on the role that lockdowns have had on student learning. These challenges too are likely to influence teachers’ perceptions of their roles as teachers. In Australia, early modelling from the Grattan Institute showed that disadvantaged students were predicted to “lose” at least one month of learning: representative of three times the loss expected of other students (Sonneman and Goss 2020). Engzell et al. (2021) found evidence of “learning loss” equivalent to one-fifth of the school year in a representative sample of 350,000 Dutch students, despite the Netherlands experiencing a relatively short lockdown and world-leading broadband access. Interestingly, in the Australian state of New South Wales, Gore and colleagues’ (2021) recent analyses of Year 3–4 mathematics and reading data across 113 schools showed no systematic decline in 2020 performance relative to 2019. However, consistent with Grattan Institute projections, Year 3 students from schools with low socioeconomic advantage showed two months less growth in 2020 than equivalent schools (Gore et al., 2021). Moreover, students themselves reported concerns about motivation, teacher support, and loneliness when learning from home, with 62.6% of adolescents believing the pandemic had negatively impacted their education (Li et al., 2021). Perhaps contributing to this problem, teachers across several studies and countries identified a lack of personal contact with students and an inability to provide individualised support as challenges to both their relationships and student learning (Phillips et al., 2021; Woltran et al., 2021).

Taken together, emerging evidence focusing on the impacts of school closures due to COVID-19 suggests significant stressors for teachers and learning risks for students. Notably, these come at a time when existing pre-COVID challenges to Australian teachers are already well documented, including significant workload challenges and administration demands, declining perceptions of autonomy, and high rates of stress (see Fray et al., 2022; Heffernan et al., 2019; Mackenzie, 2007). Drawing on the Job Demands-Resources model, therefore, there is emerging evidence of increasing pressure at a time when demands are already high. To add to this emerging empirical picture, we asked Australian teachers two broad questions about the changes to their roles at the beginning of the first COVID-19 lockdown. Teachers’ perspectives on these changes are critical for two reasons: first, they are experts within education and can offer important insights for policy and practice (Gozali et al., 2017), both during current lockdowns and afterwards, and second, the heaviest burden of remote learning arrangements fell on teachers themselves. To better understand these perspectives, we therefore sought teachers’ positive and negative perceptions of the changes to their roles. Our questions were intentionally broad, focused on “teaching roles” rather than remote learning itself, in order to capture all aspects of their role that teachers found beneficial or challenging: pedagogical, technological, relational, and practical. While our study was largely exploratory and pragmatic, with the use of teacher voice considered important for uncovering themes and perspectives that may not yet be known, our a priori expectations drawn from emerging COVID-19 research were threefold.

First, given the sheer scale of change required to convert all teaching to remote learning and the emerging findings of potential stress and exhaustion (MacIntyre et al. 2020; Silva et al., 2021; blinded), we expected Australian teachers to express concerns about additional workload. As an exacerbating factor, given findings that 75% of Australian teachers already considered their workloads unmanageable before the pandemic (Heffernan et al., 2019), we also expected teachers to express concerns about the quality of education they would be able to provide with the time and resources available to them. In a German survey conducted during school closures in May–June 2020, for example, König et al. (2020) found large differences in 89 early career teachers’ relational and pedagogical strategies for engaging and supporting students. Despite assumptions that such teachers would be “digital natives” (see Prensky 2001), not all had sophisticated digital skills and not all had access to equivalent online learning resources (König et al., 2020). For such teachers, the workload burden might be particularly strong.

Second, and consistent with emerging research in Australia and internationally, we expected teachers to report concerns related to student learning and wellbeing. When asked by Flack et al. (2020) about their top three concerns for students, approximately half of Australian teachers reported concerns about social isolation and learning loss. Concerns about student learning may be well founded: research has shown that even students who frequently use digital technologies for social or recreational reasons at home may not yet be proficient digital learners (Drane et al., 2021; Wang et al., 2014). Given that school closures had the potential to disproportionately affect students from vulnerable backgrounds, we also considered it possible that teachers would express particularly strong concerns about the learning and wellbeing of at-risk students. In Australia, for example, 13.2% of families lack internet access (Drane et al., 2021). There is also emerging evidence of mental health impacts of school closures for children and young people with mental health needs, or with additional educational needs, who may depend on school routines, relationships, and resources to support their coping (Lee, 2020; Li 2021).

Finally, we were interested to know if teachers perceived any unexpected benefits in the changes to their teaching roles as a consequence of COVID-19. Notwithstanding the multiple challenges that may have arisen when rapidly shifting to online and remote teaching, disruption may also have brought new opportunities. In emerging Australian research, for example, some school leaders identified instances in which teachers had experienced positive growth as a result of their rapid changes to teaching (Fray et al., 2022). While these cases may be exceptions, it is also possible that teachers might experience both challenges and benefits simultaneously. Within the field of technology and design, disruptions due to the unavailability of particular resources or the changing needs of end-users can catalyse processes of innovation and adoption of more efficient or effective approaches (Millar et al., 2018). Drawing on this analogy, we aimed to understand if teachers had seen any changes necessitated by remote learning as more effective than their current practice and to capture the prevalence of such responses as they arose.

## The current study

The aim of the current study was to explore teachers’ own positive and negative experiences during the implementation of remote and home-based learning during the first wave of COVID-19. While COVID-19 has now been empirically associated with additional stress and exhaustion for teachers, particularly related to work-life balance, technology issues, and concerns for students (Flack et al., 2020; Silva et al., 2021), it is important that educational leaders, policymakers, and researchers also understand what teachers themselves considered the biggest challenges in their roles when transitioning to remote learning. Such insights have implications for the future, with subsequent COVID-19 waves, climate change, and other natural disasters threatening to disrupt education indefinitely and with a changing, potentially blended landscape on the educational horizon. Given the potential for disruption to lead to innovation, we also wanted to know if teachers perceived any unexpected positives for their roles: either when teaching remotely as a result of the COVID-19 lockdown or when considering the potential for new ways of thinking about teaching when COVID-19 restrictions were lifted. Adopting both phenomenological and pragmatic perspectives, we expected the use of teacher voice to complement and extend on recent research that has investigated teacher stress and exhaustion using psychometric scales.

## Materials and methods

### Design

Our study employed a concurrent mixed-methods approach, with qualitative and quantitative data collected simultaneously (Creswell, 2009; Creswell & Plano Clark, 2007). Our primary data was qualitative, with two open-ended questions used to explore themes in teachers’ perceptions of their roles and experiences of teaching remotely during lockdown. The first question asked participants how their day-to-day role as a teacher had changed due to COVID-19, and the second question asked their perceptions of the change to remote learning during COVID-19. Given our interest in authentically representing a range of expected and unexpected teacher perspectives, responses were coded using inductive thematic analysis. To further support our interpretations of teachers’ perspectives, we also preceded each qualitative question with a shorter quantitative question: asking teachers to rate how much their role has changed and how much they preferred their former (face-to-face) or current (remote) teaching roles on a 5-point Likert scale.

### Participants

Participants included 210 Australian teachers: 163 secondary teachers, 37 primary teachers, and 10 special education teachers. Participants ranged in teaching experience (*M* = 13.9 years, *SD* = 11.00, range of 0–58) and age (*M* = 39.67 years, *SD* = 11.14, range = 20–69), and came from multiple Australian states and territories including New South Wales (49.05%), Victoria (24.76%), Australian Capital Territory (18.10%), Western Australia (7.14%), and Queensland (0.01%). There were 131 teachers employed in the public school system, 51 in the independent school system and 28 in the Catholic system. Consistent with Australian teacher employment trends (McGrath & Van Bergen, 2017), the majority of participants were female (87.14%) and a minority were male (12.86%). No participants reported identifying as non-binary, gender fluid, or other.

Because our study was conducted during the first COVID-19 wave, shortly after an Australia-wide lockdown was announced, we conducted both recruitment and data collection online. At the time, home learning had recently been implemented for the vast majority of school students across Australia. Modified learning at school was permitted for a small proportion of students whose parents could not supervise them at home, as in the case of essential workers, with tasks to mirror those completed by students at home. Thus, while most teachers also designed and implemented curriculum from home, some may also have participated in rosters to supervise at-school students to complete the home learning tasks.

To recruit participants online, we used snowballing and targeted Facebook posts. We elected to use Facebook because international studies have shown it to be the most commonly used social media platform among teachers. While equivalent Australian data are not available, 82% of American teachers report using Facebook while just 32% use Twitter (MDR, 2018). First, we contacted the administrators of Facebook pages for Australian teachers (e.g. resource sharing pages) to request permission to advertise the study. The advertisement required potential participants to be currently employed and teaching in an Australian primary or secondary school. Second, teachers were encouraged to share the survey via their own networks.

### Measures and procedure

Following institutional ethics approval from the Macquarie University Human Research Ethics Board (no. 52020631115152), teachers were asked to read an online information and consent form and to proceed to the survey proper if they would like to take part. The information and consent form and online survey were both hosted on Qualtrics. Once the survey commenced, participants answered demographic questions about gender, age, years of teaching experience, level of Australian teacher accreditation, and teaching context. Next, participants completed measures related to burnout and self-efficacy for teaching. These measures contributed to a larger quantitative analysis (see blinded) but are not the focus of the current study and are not included here. Finally, participants were asked two question sets about their current experiences during COVID-19.

The first question set asked participants if their day-to-day role as a teacher had changed due to the COVID-19 restrictions during lockdown. Participants were asked to respond on a 5-point Likert scale where 1 = not at all, 3 = somewhat, and 5 = very much. Next, participants were asked: “*Please explain how your role has changed*”. An open-text box was provided for participants to record their response.

The second question set asked participants about their perception of the change to online and remote learning during the COVID-19 lockdown. Participants were asked to respond on a 5-point Likert scale where 1 = much prefer previous teaching and 5 = much prefer current teaching. Next, participants were asked: “*Please explain your response (e.g. consider what you might miss about your previous teaching, or be glad to leave behind, as well as what you most like or dislike about the current teaching arrangements)*”. An open-text box was provided for participants to record their response.

### Coding

To code participants’ qualitative responses, we used inductive thematic analysis. Thematic analysis is a valuable and flexible tool for analysing qualitative data because it allows patterns across multiple responses to emerge (Braun and Clarke 2006; Nowell et al., 2017). This is particularly important when the qualitative responses form part of a large dataset such as ours, in which it would otherwise be difficult to identify, organise, or report on patterns latent within the data (Nowell et al., 2017). First, all responses were read closely by both authors to familiarise themselves with the data (Braun and Clarke 2006; Nowell et al., 2017).

Next, the first author generated explicit and mutually exclusive codes for each of the two qualitative questions. In response to the first question, for example, codes related to ‘delivering content online’, ‘teaching remotely’ and ‘learning new IT programs and skills’ were all organised into an ‘online and remote instruction’ theme. Six initial themes were generated and then reviewed in collaboration with the second author. During the review process, negotiation occurred about the coding of explicit vs implicit references to workload. To ensure the data were represented without researcher assumptions or bias, it was clarified that the ‘workload’ theme would only be used where explicit statements about workload, overwork, or hours of work were made and not solely where multiple online teaching activities were listed. Negotiation also occurred around codes related to ‘online tracking of student progress’ and ‘inability to teach practical lessons’, which had initially also been included in the ‘online and remote instruction’ theme. Following negotiation, these codes were considered conceptually distinct and were developed into two new themes. All other initial themes were retained: thus, eight final themes were produced in response to the first question. In response to the second question, themes were hierarchically organised into positive and negative. Three positive and nine negative themes were produced. While this represented a large number of themes, participant responses were frequently long and multifaceted with multiple themes represented. Following revision and negotiation, each theme was determined to be conceptually distinct and was retained.

Once themes were determined, responses to each qualitative question were read again to ensure thematic saturation and coherence (Braun and Clarke 2006; Nowell et al., 2017). For both qualitative questions, the themes that had been generated during the initial coding and review process were determined to appropriately capture participants’ perspectives.

## Results

Our results are reported in two sections. First, we report how, and how much, teachers perceived their roles as having changed due to COVID-19 restrictions during the first lockdown in Australia. Second, we report participants’ preferences for their current or former teaching. In both sections we report quantitative frequency data first, followed by the qualitative outcomes of our thematic analyses.

### Self-reported changes to teaching roles during COVID-19

**Fig. 1:**
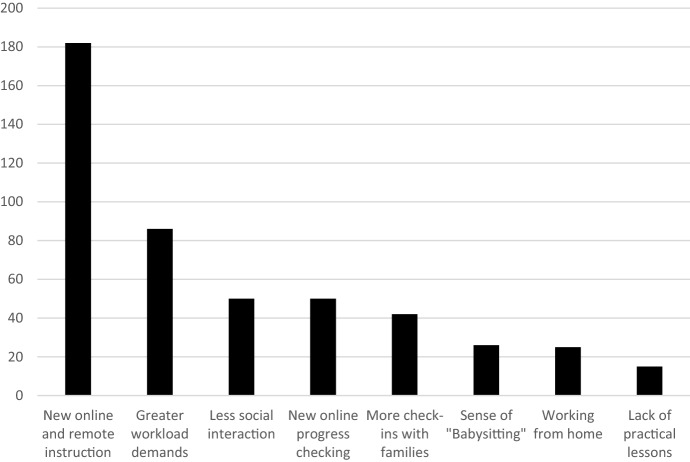
Self-reported changes to teachers’ roles as a consequence of the COVID-19 lockdown (*n* = 210)

When asked how much their current teaching roles had changed as a result of COVID-19 restrictions during lockdown, the vast majority of teachers said ‘considerably’ (13.8%) or ‘very much’ (81.0%). Just nine participants reported that their role had changed ‘somewhat’ (4.3%), while two participants said ‘not at all’ (1.0%). As shown in Fig. 1, teachers’ qualitative explanations of these changes fell into eight conceptually distinct themes.

By far the most commonly reported change in teachers’ roles related to the shift to online and remote instruction (86.7%). According to teachers this shift required multifaceted redesign, with implications for stimuli, delivery methods, learning activities, assessments, and pedagogy. For example, Lane (56, secondary teacher) described multifaceted changes to activity materials and stimuli, to intended activities, and to assessments:

*Tweaking of or completely changing PowerPoints, worksheets and activities. For example, group work to individual work… finding suitable resources such as videos to demonstrate scientific concepts or practicals…. Changing assessment tasks to online formats. Senior tasks that were to be based on excursions now have to be rewritten.*

Similarly, Fay (60, secondary teacher) described the series of questions about activities, delivery, and student accessibility that she needed to answer in order to pivot learning online in an effective way:

*Planning online learning materials is extensive and exhausting... requiring flexibility and innovation to present and deliver the learning materials in a way that is meaningful, relevant, and accessible to students. What digital platform to use? Can the students actually do those activities in their homes? Do they have access to the internet? Are they sharing devices with other family members? What resources do they have at home? How do you track what [they] are doing?*

Finally, Benjamin (34, secondary teacher) described his process of ‘rethinking’, ‘reworking’, and ‘rewriting’ in order to make this online transition and to supporting other staff to do so:

*The way I am doing things is very different… we have had to rework our scopes and sequences for stage 4-6, rethink and rewrite assessment tasks, assist my team in setting up for remote learning including the use of a brand-new online platform that they hadn’t used before, etc.*

The second most commonly reported change in teachers’ roles related to workloads (41.0%). As a consequence of the rapid shift to online and remote instruction, numerous teachers reported working extraordinarily long hours with insufficient preparation time. Zara (56, secondary teacher) stated that over the last three weeks of term she “*probably averaged five hours of sleep per night”*, for example, while Rowena (40, secondary teacher) suggested that COVID-19 had “*extended my working days to 15 h or more*” and Chantelle (55, secondary teacher) reported working “*most days from 8:30am to well after 10:00 pm*”. Bella (26, secondary teacher) stated that she was “*expected to be online, not from 9 to 3 but 24 h a day*” in order to “*respond to students, parents, caregivers, and the school executive*”.

The sharp increase in teachers’ workload had a negative impact. Sabine (42, secondary teacher) stated that her work had “*[taken] away valuable family time*”. According to Alina (33, secondary teacher), “*The workload has increased even more than I thought possible. It's soul destroying. And then when the technology keeps failing it's hard not to cry*”. Patrick (54, secondary teacher) similarly reported that he “*spend[s] hours doing work online and then responding to students and parents beyond what would be considered normal… I have a very short fuse and am quick to anger, something I never did before*”. According to Zoe (29, secondary teacher), such work would *“usually take years to develop*”.

Other less common themes emerging from teachers’ responses about changes to their current roles included more check-ins with families (“*mainly emails with parents*”; “*I’ve spent entire days calling parents”*), less social interaction with students (“*limited contact teaching time with students*”; “*feeling very isolated from my students*”), and the difficulty of checking students’ progress online (“*The joy of… being able to see their work as they go, even being able to be present to offer on the spot help/advice is gone*”). These latter two themes are also echoed in our second set of analyses and discussed in more detail below. Finally, some teachers discussed the practicalities of working from home (*“I am sitting in front of a computer with my own children distracting me”)*, a sense of “babysitting” rather than teaching (“*sometimes it’s more babysitting*”; “*I am [treated] by the government… as a ‘baby sitter’ where my health is at risk*”), and a lack of practical lessons (“*Also anything practical is near impossible given I am a science teacher*”) as ways in which their roles had changed.

### Teachers’ perceptions of teaching during COVID-19

When asked whether they prefer their previous teaching, before COVID-19, or their current teaching, during the COVID-19 lockdown, three quarters of teachers (76.2%) reported ‘preferring’ or ‘much preferring’ their previous teaching. A smaller number expressed ‘no preference’ (17.1%), while a very small number ‘preferred’ or ‘much preferred’ their current teaching role (6.2%). When asked to explain their responses, the majority of teachers reported negative themes related to their changed roles (88.6%). Interestingly, however, almost half also reported positive themes (44.8%).

**Fig. 2:**
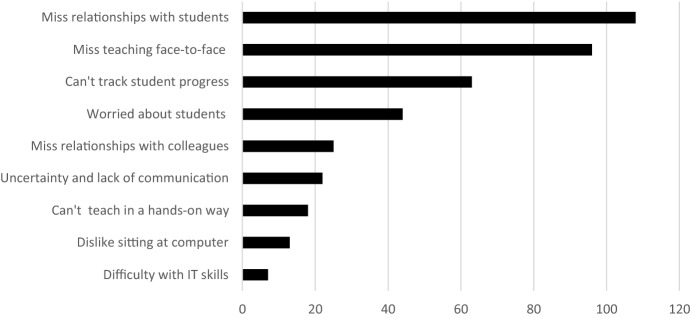
Negative themes related to teachers’ changed roles during lockdown (*n* = 210)

Across the nine negative themes outlined in Fig. 2, participants most commonly reported in their current roles that they deeply missed their relationships with students (51.4%) and teaching face-to-face (45.7%). A sizeable number also reported being unable to track student learning (30.0%) and feeling worried about student learning and wellbeing (21.0%). Thus, care and concern for students were prominent across responses. Surprisingly, concerns about the lack of hands-on pedagogy (8.6%) and issues with IT skills (3.3%) were less common. Indeed, turning to the positive aspects of their current roles, 40 teachers (19.1%) enjoyed learning new online skills and integrating IT in new ways. A further 30 reported enjoying practical aspects of working from home (14.3%), such as the lack of commute, while 29 reported that they enjoyed not needing to manage difficult student behaviours (13.8%). We discuss findings for our most common positive and negative themes below.

### Negative themes

As our most common negative theme related to their new teaching role during COVID-19, a majority of teachers (51.4%) described deeply missing their students. As 26-year-old primary teacher Rosie reported, “*I miss my students—I became a teacher for them so feel disconnected from the job with them learning remotely*”. Natalie (50, secondary teacher) said “*easy, I love my students and miss them*”, Fatima (54, secondary teacher) said “*miss the students, seniors are delightful!”,* while Tom (38, secondary teacher) explained that “*I miss the spontaneity that occurs in classrooms and the playground… And I miss kids!*”. Teachers within this theme also made reference to the relational nature of teaching as part of these responses:

*Teaching is relational and being remote makes maintaining those relationships much harder.* (Jodie, 67, secondary teacher)

*Teaching is relational. It is live and requires human interaction. We are doing our utmost to replicate but it’s draining. I’m doing more for Year 12 than ever before - without the fun bits.* (Sahara, 47, secondary teacher)

The second most common theme to emerge, in 45.7% of responses, was that teachers also missed teaching face-to-face. Frequently these responses were combined with statements about missing students, but extended beyond interpersonal statements to also include pedagogical references to student learning and engagement. Aneeka (40, primary teacher) stated that she ““*miss[ed] the kids and being able to teach face to face*”, for example, while Lilia (37, secondary teacher) stated that she… “*miss[ed] seeing students face to face and the wonderful and hilarious things they come up with… I also miss the challenge of getting to students who are struggling*”. Wilhelmina (40, secondary teacher) explained that *“face-to-face interaction is vital, not only for improved learning outcomes but for social and emotional wellbeing”.* Heyam (43, primary teacher) further explained that face-to-face teaching enabled lessons to be “*more fluid and student led*” while Maddie (28, primary teacher) explained that “*face-to-face teaching enables you to focus specifically on the students’ needs minute-by-minute as they are in front of you*”.

As part of their explanations regarding face-to-face teaching, several teachers mentioned the joy of observing student learning directly. Jessica (36, primary teacher) explained that she “*miss[ed] the connection with the students I have from face-to-face teaching and those light bulb moments when students click*”, while Maryanne (32, secondary teacher) similarly reported: “*I miss seeing all my students face to face… the incidental teaching and light bulb moments are the reason I love my job*”. As demonstrated in the responses of Sammi and Michael below, such moments were important both for motivating teachers and for supporting appropriate pedagogical responses:

*I sorely miss seeing their smiling faces and the noise of learning in my classroom. I miss the grateful hugs I receive at the end of each day and the looks of amazement when they learn something new or perfect a new skill. I miss being able to show I am proud of them in person and give them the face to face contact they need at a young age.* (Sammi, 24, secondary teacher)

*I miss the instantaneous ability to recognise cognition, to treat it in the moment and make adjustments. Seeing the light go on or the blank stare is easier to rectify than being bombarded with 23 emails asking me to explain parts a,b, or c*. (Michael, 45, secondary teacher)

A third and related theme that emerged in teachers’ responses regarding their changed teaching roles was about the difficulty of tracking student progress during lockdown (30.0%). Susan (67, secondary teacher) stated that she “*can’t really see if students understand, and following up when students don’t attend live lessons or hand in work is very time consuming*”, while Julia (33, primary teacher) explained that she “*hate[s] not knowing where my kids are at academically and I am yet to find a way to accurately assess anything in these trying times*”. In some cases, teachers reported specific concerns about students not submitting work in the first place. Joy (64, secondary teacher) stated that “*there is no way of telling if students are completing the work set for them*”, while Rikki (47, primary teacher) similarly stated: “*It’s very hard to monitor the progress of some students as they don’t complete many of the online activities*”. Erin (56, secondary teacher) explained that there was “*additional work chasing up students who are absent or not submitting work for feedback*” or who were “*having difficulties with the technology or the internet dropping out*”. Overall, as demonstrated by the responses of Sarah, Jamie, and Cath below, teachers expressed a sense of helplessness about this situation:

*I am finding it difficult to connect with all my students. If they don’t engage in the classroom I can always try and help them. If they don’t engage online (if they don’t even log on) there is nothing I can do*. (Sarah, 52, secondary teacher)

*I miss the children, the hugs and high-fives… I can't see what they are doing at home and some haven't engaged at all with online learning, despite phone calls to parents, lending devices, zoom sessions, etc. *(Jamie, 46, primary teacher)

*I can’t call home to discuss disengaged learners because we’re being sensitive to potential issues at home, i.e. job loss. A lot of students are falling through the cracks… But I can’t call and talk to them. A lot of students don’t have internet or 4G at home so I literally haven’t heard from them since mid-March. *(Cath, 25, secondary teacher)

Fourth, many teachers reported concerns about students’ wellbeing (21.0%). For example, Rachel (24, primary teacher) reported being “*a lot more worried now about my students who need personal contact to alleviate wellbeing concerns”*, while Phoebe (25, secondary teacher) stated that she would be “*glad to be able to walk back into my Year 12 classroom and be able to support my students rather than have them fear about their future and their HSC”*. Teachers were keenly aware of students’ potential stressors and emotional states and frustrated that they were unable to help:

*I'm really hating not being about to have little one on one chats with kids, just to check they are OK. And I'm not liking the looks of loneliness, frustration, depression, resignation creeping onto some students' faces… It’s heartbreaking that I only have empty words to give to them. *(Yasmin, 47, secondary teacher)

*Students who suffer complex trauma or have additional learning needs are those who make the face to face teaching the most challenging and rewarding - and they aren't online. I worry about them. *(Alexandra, 46, secondary teacher).

Finally, and in addition to missing their colleagues (11.9%), a significant minority of teachers reported finding continually changing information about lockdown arrangements difficult (10.5%). Rod (26, secondary teacher) suggested that “*there are a lot of sudden changes, poor communication of plans, lack of consistency with the decisions being made, and expectations set at a wider level (basically every level above principal right up to the… PM)*” while Harriet (40, secondary teacher) explained that “*lack of clear direction from politicians has meant a lot of extra work for teachers already struggling to change their whole teaching program*”. Jasper (38, secondary teacher) felt that there were changes “*every 5 min at the whim of politicians… the amount of time, energy and pressure to do a single lesson has increased exponentially.*” Importantly, teachers identified that their sense of disenfranchisement was particularly strong when their own commitment and hard work was unrecognised. This was evident in the responses of Ruth and Bella below:

*I do not like constantly having the bar moved by politicians with absolutely no consultation or respectful dialogue or even respectful monologue. I have been given little choice about my… safety within the workplace. I have been belittled by the Prime Minister and the Premier in increasingly personal attacks about my profession.* (Ruth, 34, secondary teacher)

*I am now having to defend myself and my role as a teacher because everyone thinks the schools are closed (THEY NEVER CLOSED) and that teachers are at home having the time of their life. This is f#%d. *(Bella, 26, secondary teacher)

Together with less common themes about sitting in front of a computer, difficulties with IT, and not teaching in a hands-on manner, these diverse and multifaceted concerns suggest teachers faced a range of difficulties related to their new roles during the shift to online and remote learning during COVID-19.

### Positive themes

An important finding of this study was the emergence of positive themes alongside challenges. Indeed, some teachers reported explicitly seeking such reflection as part of their changing role (“*I keep looking for positives in this experience though… such as improved formative assessment.”*). Although the workload associated with the shift to online and remote teaching was challenging, for example, the most commonly reported positive theme was that many teachers enjoyed the development and application of new IT skills. Teresa (35, secondary teacher) liked “*learning new technical skills to pass onto students*”, while Mera (29, secondary teacher) saw benefits for the school community:

*The silver lining is when the all is over, more people in my workspace will understand ICT and technology better, meaning less resistance to change for plans I wish to implement (online document collaboration, cloud file sharing etc).*

Bess (35, secondary teacher) also stated that she loves “*the ease of adding links and assistive technologies, such as text-to-read videos that assist in explaining concepts and topics*”, while James (61, primary teacher) saw value in learning about particular applications:

*There are some aspects of my current teaching that I enjoy… the online apps and resources that I am learning about that we could use if we return to our previous teaching methods. E.g. Screencastify, Google Earth, Clips, Minecraft, ClickView, Google Classroom etc.*

Two additional positive themes related to teachers’ changed roles also emerged. Some teachers also saw practical benefits of working from home, such as no commute, while others also reported enjoying not needing to manage difficult student behaviours. Samantha (24, secondary teacher) said she was “*glad to leave behind the behavioural issues I have to deal with on a daily basis*”, while Xanthea (39, secondary teacher) reported that it had been “*a welcome challenge to be able to create innovative and engaging lesson content without the concern of behaviour management.*”. As exemplified by Jacinda (41, alternative education teacher), however, these statements often sat alongside concerns about these same students:

*Not going to pretend that it’s all bad. It is nice getting a bit of a break from the aggressive and very difficult students (many are still in but are having a day off here and there) but our students need one to one support and assistance.*

## Discussion

The aim of this study was to explore teachers’ own positive and negative experiences during the implementation of remote and online learning during the first wave of COVID-19. We used the Job Demands-Resources model to better understand the demands placed on teachers within particular roles and the cumulative impacts of different multifaceted demands on functioning (Demerouti et al., 2001), and we drew on both pragmatic and phenomenological paradigms when selecting our self-report methodology. Teachers were asked two key questions: one about changes to their teaching role as a result of restrictions during the first COVID-19 lockdown and one about their preferences for teaching previously, prior to COVID-19, or currently, during COVID-19. To ensure the strong presence of teacher voice, and to enable patterns across teacher responses to emerge (Braun and Clarke 2006), qualitative explanations were coded using inductive thematic analysis.

In response to the first question, teachers described comprehensive and multifaceted changes to their teaching activities including lesson design and materials, pedagogy, feedback, and assessment. As predicted, these changes had strong workload implications. Explicit in teachers’ responses was that the sheer volume of work was relentless and unsustainable, with negative impacts on family time and their own mental health. While these findings are consistent with findings of teacher stress and exhaustion in international samples (MacIntyre et al. 2020; Silva et al., 2021; Sokal et al., 2020; blinded), they offer additional insights into the workload demands that teachers themselves considered most challenging. Interestingly, and counter to our predictions, self-reported concerns regarding technology and technological self-efficacy were low (cf. Haverback 2020; Košir et al. 2020). Indeed, in response to our second question, many teachers reported that they valued the opportunity to learn and implement new IT skills, platforms, and programs. What emerged instead were structural concerns about work intensification that was beyond even the most efficacious teachers’ control, and yet necessary in order to support students and their learning. Drawing on the Job Demands-Resources model, there was a perception that the current job demands were not manageable and could not be mitigated with personal resources alone. Further, from teachers’ perspectives, this intensification was also made worse by sudden changes in demands from government and by a harmful false rhetoric that their roles had been made easier and not harder by the lockdown. It also had implications for their mental health.

In response to the second question, the vast majority of teachers reported negative themes related to their current roles during lockdown: particularly related to missing students and face-to-face teaching. That more than half the participating teachers explicitly described missing their students, and their relationships with students, highlights the critical importance of the student–teacher relationship for teacher wellbeing. Teachers reported having close emotional bonds with their student cohorts and feeling lost without regular student contact. While teaching has previously been described as a type of emotional or relational labour (McGrath & Van Bergen, 2019; Van Bergen et al. 2018), it also has important emotional benefits. Consistent with the notion of relational pedagogy, and with past research on the benefits of a positive student–teacher relationships and support (Burns et al., 2019; Hamre & Pianta, 2001; Hughes, 2011; McGrath & Van Bergen, 2015), teachers also highlighted how such relationships were important for student learning.

Only one other study to our knowledge has identified the prevalence of these same relational concerns among teachers. When asked about issues they were struggling with and needed support with, 18 of 64 participating teachers (predominantly from Australia, New Zealand, Singapore and the United States) identified connectivity with students and families as an issue (Phillips et al., 2021). Concerns about connectivity included both practical issues related to internet connectivity and communication, and relational issues related to social connection. While this prevalence is lower than the majority of teachers who identified missing their relationships in the current study, we note the potential for the different question wording to also drive responses: in our study, we asked about why educators preferred teaching previously or currently, whereas Phillips and colleagues (2021) asked teachers to identify issues they needed support with. It is possible that some teachers were affected emotionally by the relational distance they were experiencing from their students, but did not see immediate or practical remedies to this problem. Supporting this possibility, Letzel et al., (2020, p.166) suggest their findings of teacher anger and nervousness during lockdown may also be partially explained by teachers missing students. Taken together, these studies show the importance of asking teachers specifically about both preferences and challenges in their roles before and during lockdown. They also highlight a specific risk to teacher retention and wellbeing that is not solely accounted for by workload concerns.

As predicted, and as highlighted in emerging research internationally (e.g. Phillips et al. Woltran et al., 2021), teachers also reported difficulty tracking students’ progress and meeting students’ wellbeing needs during lockdown, with implications for the education they were able to provide. These concerns often centred on students who were not logging on or submitting work, who did not have the technological resources to engage effectively, or who were emotionally sensitive or vulnerable. Concerns about the differential impacts of lockdown on specific students are supported by Gore and colleagues’ (2021) recent findings that slower learning growth across the first COVID-19 lockdown was concentrated in students from schools with low socioeconomic advantage, and by Li and colleagues’ (2021) findings that many students themselves felt it more difficult to access teacher support when learning remotely. Given their keen awareness of the students who may be most at risk, when, and in what ways, it critically important that teachers be included in discussions about the provision of intensive educational and wellbeing support for students who need it.

Finally, while less frequent than negative themes, our finding that almost half the teachers reported one or more positive themes related to their adapted teaching roles during lockdown suggests both opportunities for growth and insights into the day-to-day stressors facing teachers in the classroom. Not surprisingly, given that classroom misbehaviour is a key emotional stressor for teachers (McGrath & Van Bergen, 2019), teachers reported enjoying the reduction in classroom management. However, there were also pedagogical benefits related to the shift to online teaching itself. Disruption brings with it both challenges and opportunities, with the potential to catalyse new efficient or effective approaches (Millar et al., 2018). Consistent with this notion, a large number of teachers reported that they had enjoyed learning new technological skills and using new programs. Several reported a desire to bring these learnings into the face-to-face classroom.

### Implications

Our study has implications for current supports provided to teachers, for online and remote teaching broadly, and for future emergency planning. As a matter of workplace health and safety, for example, we highlight the strong need for accessible mental health services for teachers, for future pandemic planning that centralises at least some aspects of online lesson design and resource allocation, and for government communication which acknowledges the extraordinary contributions teachers have made across the life of the COVID-19 pandemic (see Fray et al., 2022). While future lockdowns are dependent upon case numbers, vaccinations, and local policies, there remains a possibility of further restrictions as new COVID-19 variants emerge. We expect the benefits of this future planning to extend beyond COVID-19 to natural disasters, including floods and bushfires.

Given the barrier that some teachers encountered when trying to check in on students who did not have sufficient technology access, we also highlight the need for national conversations about educational equity and the provision of learning resources at home (see Drane et al., 2021). Finally, given that 21% of teachers were concerned for students’ stress, anxiety, and wellbeing during lockdown, we highlight the importance of accessible paediatric and youth mental health care screening to mitigate against these potential concerns (see Li et al., 2021). We suggest a role for government policy and funding in supporting these responses so as to remove some burden from teachers.

### Limitations and future directions

There are three methodological limitations of the current study. First, our methodology can only capture explicit themes that emerge in teachers’ written responses. For this reason, our findings should be seen as representing the most salient issues and concerns reported by teachers when considering their changed roles, and not all those they agree with. Second, given our online recruitment, our findings may not be representative of all Australian teachers. Third, we note that greater concerns about excessive workloads emerged in response to our first question, about how teachers’ roles had changed due to COVID-19, while the sheer extent of teachers’ care for their students only emerged in response to the second question. We suggest that the first question was seen by teachers as seeking structural or factual information, while the second was seen as seeking personal preferences. Given how sensitive teachers were to question wording—a finding supported by the diverse challenges and stressors reported in other published studies using distinct question sets—we recommend that future research about pandemic impacts continue to employ multifaceted questioning to ensure teachers views are fully represented.

Over and above these limitations, we highlight a role for future research to track teachers’ perspectives of lockdown and of other educational disruptions over time, across different genders, career stages, and jurisdictions with different schooling provisions. Given the emergence of strong relational themes, with the majority of Australian teachers reporting missing their students, we also recommend future research that examines these responses according to teachers’ own motivations for teaching and provision of student support. While there are calls to preserve the physical school-space following COVID-19, there are also predicted increases in blended learning and a potential risk that public education could come to rely on digital platforms and content created by private companies (UNESCO, 2020). Our relational themes may be particularly important when considering how best to reshape education into the future. Finally, we recommend that future research considers teacher stress and exhaustion in light of both the job resources identified in the literature, such as self-efficacy (Haverback 2020; Košir et al. 2020), and the job demands identified by teachers themselves, such as workload constraints.

## Conclusion

This study provided insight into Australian teachers’ perceptions of their roles as teachers during the first COVID-19 lockdown. The findings support and extend the findings of recent research in other jurisdictions, including research focussed on teachers’ perceptions of the changes required to instructional design (Flack et al., 2020; König et al., 2020), their technological and instructional self-efficacy (Haverback 2020; Košir et al.2020), and teacher and student mental health and wellbeing (Košir et al. 2020; MacIntyre et al. 2020; Sokal et al., 2020; Silva et al., 2021). While almost half (44.8%) of the Australian teachers who took part in the research identified potential benefits in their changed roles due to lockdown, such as the development of new technological skills and approaches, the vast majority also reported challenges with excessive workload, with missing their students, and with tracking students’ progress online. We highlight possible implications for education systems and government in supporting teachers and students as they return to face-to-face learning.
